# Clinical, functional, and computed tomographic characterization of idiopathic inflammatory myopathy-associated interstitial lung disease: a retrospective cohort study

**DOI:** 10.36416/1806-3756/e20250123

**Published:** 2025-09-22

**Authors:** José Ricardo Bandeira de Oliveira, André Nathan Costa, Bruno Guedes Baldi, Mark Wanderley, Marcio Valente Yamada Sawamura, Ronaldo Adib Kairalla

**Affiliations:** 1. Divisão de Pneumologia, Instituto do Coração - InCor - Hospital das Clínicas, Faculdade de Medicina, Universidade de São Paulo - HCFMUSP - São Paulo (SP) Brasil.; 2. Instituto de Radiologia, Hospital das Clínicas, Faculdade de Medicina, Universidade de São Paulo - HCFMUSP - São Paulo (SP) Brasil.

**Keywords:** Lung diseases, interstitial, Myositis, Antisynthetase syndrome, Tomography, Spirometry

## Abstract

**Objective:**

To evaluate clinical, functional, and CT characteristics, as well as disease progression, in patients with idiopathic inflammatory myopathy-associated interstitial lung disease (IIM-ILD) treated at a referral center in Brazil.

**Methods:**

This was a retrospective cohort study analyzing demographic characteristics, clinical variables, pulmonary function test results, HRCT findings, serological profiles, treatments, and outcomes.

**Results:**

Seventy-nine IIM-ILD patients were included in the present study. The mean follow-up period was 8.7 ± 4.7 years. The most common diagnosis was antisynthetase syndrome, observed in 51 (64.5%) of the 79 patients. The most common symptoms were dyspnea (in 94.9%), arthralgia (in 82.2%), and muscle weakness (in 75.9%). Mean baseline FVC was 2.19 ± 0.75 L, corresponding to 62.5% of the predicted value. During follow-up, FVC showed significant improvement. The most common CT patterns were indeterminate (in 44.4%) and nonspecific interstitial pneumonia (in 35.4%). Treatment most frequently included prednisone (in 98.7%), azathioprine (in 92.3%), or methotrexate (in 57.7%). Overall survival was 84.8%. Mortality was higher among patients who developed pulmonary hypertension and those who required intravenous methylprednisolone pulse therapy.

**Conclusions:**

Most patients with IIM-ILD progress well with immunosuppressive therapy. Pulmonary hypertension and the need for methylprednisolone pulse therapy appear to be associated with higher mortality.

## INTRODUCTION

Idiopathic inflammatory myopathies (IIMs) are systemic autoimmune disorders that primarily affect skeletal muscle and often involve interstitial lung disease (ILD), a major contributor to morbidity and mortality. Antisynthetase syndrome (ASyS) is a rare IIM subtype that is defined by anti-aminoacyl-tRNA synthetase (anti-ARS) antibodies and features such as myositis, ILD, arthritis, Raynaud’s phenomenon, and mechanic’s hands.^1^ Although ASyS was initially classified as an IIM subtype, it may present as isolated ILD, especially in patients with anti-PL-7 or anti-PL-12 antibodies.^2^ Several IIM-related autoantibodies aid in diagnosis and stratification. Among anti-ARS antibodies, anti-Jo-1 is the most common and is strongly associated with ILD, which may precede myositis in up to 20% of cases.^3^ ILD affects 23-65% of IIM patients and is the leading cause of mortality in this group.^1^ ^,^ ^4^ The pathogenesis of IIM-associated ILD (IIM-ILD) is unclear but likely shares initial immune mechanisms with muscle involvement.^5^ IIM-ILD has a highly variable course, from stable disease to rapidly progressive forms with marked lung function decline and reduced quality of life.^6^ The Bohan and Peter classification, established in 1975, remains a cornerstone in the diagnosis of IIM. It proposes five key criteria: symmetric proximal muscle weakness; elevated serum muscle enzymes; myopathic changes on electromyography; characteristic muscle biopsy findings; and typical skin rash (heliotrope rash or Gottron’s papules) for dermatomyositis. A diagnosis is categorized as definite, probable, or possible polymyositis or dermatomyositis depending on the number and combination of criteria met. Despite limitations related to specificity and overlap syndromes, the Bohan and Peter framework continues to be widely referenced in clinical and research settings.^1^ ^,^ ^4^ Early diagnosis and treatment are critical for improved outcomes, but management remains a challenge because of the limited data available from clinical trials and the lack of specific ILD guidelines for IIM/ASyS.^7^ The objective of the present study was to evaluate clinical, functional, and CT characteristics, as well as disease progression, in patients with IIM/ASyS treated at a referral center in Brazil. 

## METHODS

This was a retrospective observational cohort study including IIM/ASyS patients followed at an ILD referral center in Brazil between 1986 and 2020. Diagnosis was based on Bohan and Peter criteria (≥ 2 for probable myositis) or positive anti-ARS antibodies. Adults with ≥ 2 pulmonary function tests were included to allow longitudinal analysis. Data from 79 of 132 IIM patients were analyzed; the remaining patients had missing information and were therefore excluded from the analysis. Demographic, clinical, radiological, functional, and laboratory data were collected via a thorough review of electronic and paper medical records. Variables included age, sex, time to diagnosis, extramuscular features, serology (for anti-ARS antibodies), and treatments. Primary outcomes were functional decline, as assessed by pulmonary function tests, and overall survival. Patients were followed from the diagnosis of ILD until death or their last documented visit. Treatment-related adverse events were also recorded. Because of the noninterventional nature of our study, with no additional risk to patients and no intervention other than routine outpatient follow-up care, a waiver of written informed consent was requested on the basis of Brazilian National Health Council Resolution no. 466/2012. The study was approved by the local research ethics committee (Protocol no. 2.827.565). 

Categorical variables were reported as frequencies, whereas continuous variables were reported as mean ± SD or median [IQR], depending on their distribution. Values of FVC and percent predicted FVC (FVC%) were described as mean ± SD and median [min-max]. Paired t-tests were used in order to compare baseline and final FVC values. Survival was analyzed by the Kaplan-Meier method. The level of significance was set at p < 0.05. All analyses were performed with the IBM SPSS Statistics software package for Windows, version 21.0 (IBM Corporation, Armonk, NY, USA). 

## RESULTS

Of a total of 132 IIM patients, 79 (59.8%) met the inclusion criteria. Most (75.9%) were female, with a mean age of 45.2 ± 13.2 years (range, 18-82) and a mean follow-up of 8.7 ± 4.7 years. ASyS was the most common phenotype, in 51 (64.5%) of the 79 patients included in the study (Table 1). 

**Table 1:** Clinical, demographic, laboratory, and functional variables in patients with idiopathic inflammatory myopathies.^a^

Variable	N = 79
Age at diagnosis	
Age, years	45.2 ± 13.2
Sex	
Female	60 (75.9)
Diagnosis	
Antisynthetase syndrome	51 (64.5)
Amyopathic dermatomyositis	12 (15.1)
Dermatomyositis	08 (10.2)
Polymyositis	06 (7.6)
Anti-MDA5 syndrome	01 (1.4)
Anti-PM/Scl syndrome	01 (1.4)
Positivity for IIM-related autoantibodies	
Anti-Jo-1	46 (58.2)
Anti-PL-12	02 (2.5)
Anti-PL-7	02 (2.5)
Anti-PL-12 and anti-Jo-1	01 (1.2)
Anti-PM/Scl	01 (1.2)
Anti-MDA5	01 (1.2)
Time from symptom onset to diagnosis (n = 67)	
Time elapsed, months	17.7 ± 28.9
Dyspnea at diagnosis (n = 79)	75 (94.9)
mMRC scale score	
0	2 (2.5)
1	13 (16.4)
2	17 (21.5)
3	24 (30.3)
4	23 (29.3)
Clinical manifestations (n = 79)	
Arthralgia	65 (82.2)
Proximal muscle weakness	60 (75.9)
Cough	56 (70.8)
Mechanic’s hands	55 (69.6)
Myalgia	49 (62.0)
Raynaud’s phenomenon	48 (60.7)
Weight loss	48 (60.7)
Gastroesophageal reflux disease	47 (59.4)
Fever	37 (46.8)
Dysphagia	33 (41.7)
Drugs	
Oral prednisone	77/78 (98.7)
Oral azathioprine	72/78 (92.3)
Oral methotrexate	45/78 (57.7)
Intravenous methylprednisolone pulse therapy	33/77 (42.9)
Oral cyclosporine	32/78 (41.0)
Oral mycophenolate mofetil	31/76 (40.8)
Intravenous cyclophosphamide pulse therapy	29/78 (37.2)
Rituximab	19/77 (24.7)
Primary cancer	
Lung	02 (16.7%)
Cervix	02 (16.7%)
Stomach	02 (16.7%)
Salivary gland	1 (8.3%)
Leukemia	1 (8.3%)
Lymphoma	1 (8.3%)
Breast	1 (8.3%)
Melanoma	1 (8.3%)
Prostate	1 (8.3%)

IIM: idiopathic inflammatory myopathy; and mMRC: modified Medical Research Council. ^a^Data expressed as n (%) or mean ± SD.

The mean time from symptom onset to diagnosis was 17.7 ± 28.9 months; n = 67). Dyspnea was the most common symptom (in 94.9% of the 79 patients included in the study). In most cases, dyspnea was classified as severe (a modified Medical Research Council scale score of 3 in 24 patients [30.3%] and of 4 in 23 [29.3%]). Symptomatic improvement in dyspnea was seen in 59 of 70 patients (84.2%), most commonly after initiation of pharmacological treatment. Cough was present in 56 (70.8%) of the 79 patients, and 38 (48.2%) reported an improvement in their cough after treatment, whereas 10 (12.6%) had no improvement. The most common extrapulmonary manifestations were arthralgia (in 82.2%), muscle weakness (in 75.9%), and mechanic’s hands (in 69.6%); symptoms such as fever, weight loss, and Raynaud’s phenomenon were also common. When available, data were analyzed and, in general, immunosuppressive treatment led to an improvement in the symptoms of arthralgia in 44 patients (77.2%; n = 57); muscle weakness in 54 patients (100%; n = 54); and mechanic’s hands in 46 patients (88.4%; n = 52). 

Antinuclear antibody was positive in 65.8% (50/76), with titers ≥ 1/320 in 44%; the cytoplasmic pattern was the most common (50%; n = 76). Rheumatoid factor was positive in 20.8% (14/67), and anti-cyclic citrullinated peptide antibody was positive in 28.5% (4/14). Anti-SSA was found in 30.4% (21/69), and anti-SSB was found in 4.3% (3/69). Among anti-ARS antibodies, anti-Jo-1 was the most common (in 55.3%), followed by anti-PL-12 (in 9.1%) and anti-PL-7 (in 3.9%; Table 1). Corticosteroids were used in 98.7% (n = 78) of patients, typically combined with azathioprine or methotrexate. Treatment was individualized and adjusted on the basis of clinical response and adverse events. Intravenous methylprednisolone was given to 42.9%, and intravenous cyclophosphamide was given to 37.2%. Mycophenolate mofetil was used in 40.7%, and methotrexate was used in 57.7%. Azathioprine was associated with adverse events (including nausea, cytopenia, and liver enzymes) in 63.8%, and methotrexate was associated with adverse events in 26.6%, with one case of interstitial reaction (Table 1). 

**Figure 1:**
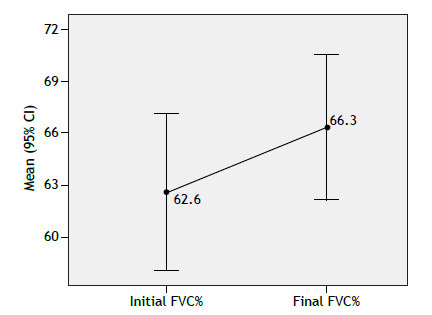
Comparison of percent predicted FVC (FVC%) values between the first and last assessment in 79 patients with idiopathic inflammatory myopathies.

Patients underwent a mean of 5.9 spirometry tests (n = 79). As can be seen in Table 2, initial FVC was 2.19 ± 0.75 L, and FVC% was 62.6 ± 20.3%. DL_CO_ (in % of predicted) was available for 27 patients, averaging 50.6 ± 19.3%. FVC decreased by ≥ 10% in 25.3%, improved by ≥ 10% in 35.4%, and remained stable in 39.3% (Figure 1). Overall, FVC% improved over time, with no association between outcomes and antibody subtype or treatment. 

**Table 2:** Comparison of FVC values (in L and % of predicted) between the first and last assessment in 79 patients with idiopathic inflammatory myopathies.^a^

Variable	Initial	Final
FVC, L	2.19 ± 0.75	2.26 ± 0.79
FVC, % predicted	62.6 ± 20.3	66.3 ± 18.3

a Data expressed as mean ± SD.

All 79 patients underwent HRCT. Initial scans showed minimal changes, with interstitial lung abnormalities in only one patient (1.3%). As can be seen in Table 3, the most common CT pattern was indeterminate (in 44.4%), followed by nonspecific interstitial pneumonia (in 35.4%) and nonspecific interstitial pneumonia + organizing pneumonia (in 13.9%). Mean pulmonary artery trunk diameter was 2.78 ± 0.37 cm, and the pulmonary artery trunk/aorta ratio was 0.91 ± 0.11 (i.e., not suggestive of pulmonary hypertension). 

**Table 3:** CT patterns observed in patients with idiopathic inflammatory myopathies.^a^

CT pattern	N = 79
INDETERMINATE	35 (44.4%)
NSIP	28 (35.4%)
NSIP + OP	11 (13.9%)
UIP	2 (2.5%)
OP	2 (2.5%)
ILAs	1 (1.3%)

ILAs: interstitial lung abnormalities; NSIP: nonspecific interstitial pneumonia; OP: organizing pneumonia; and UIP: usual interstitial pneumonia. ^a^Data expressed as n (%).

The most common comorbidities were hypertension (in 41.7%), dyslipidemia (in 39.2%), and diabetes (in 17.7%). Malignancy was reported in 15.1% (n = 12), with the lungs, cervix, and stomach being the most common primary sites (two cases each; Table 1). Echocardiograms were performed in 79.7% (n = 63); 23.8% showed pulmonary artery systolic pressure > 35 mmHg or tricuspid regurgitant jet velocity > 2.7 m/s, being suggestive of pulmonary hypertension. Right ventricular dysfunction was found in only 1 patient, who required high-flow oxygen at initial evaluation. 

**Figure 2:**
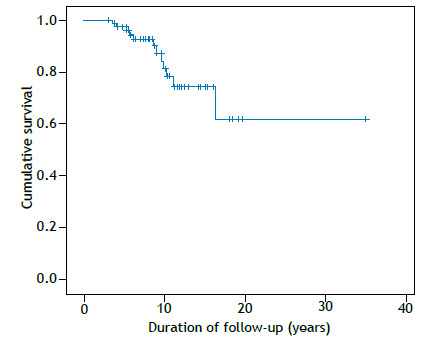
Cumulative survival in 79 patients with idiopathic inflammatory myopathies and interstitial lung disease.

**Figure 3:**
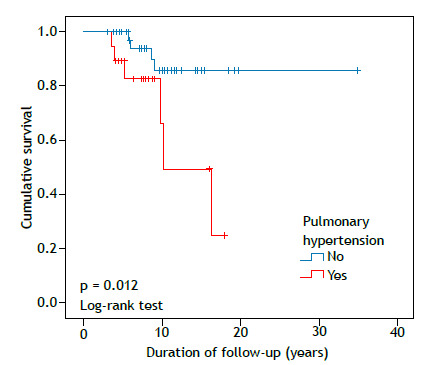
Survival curves according to the presence or absence of pulmonary hypertension on echocardiography.

Mean overall survival was 25.9 years (95% CI, 20.6-31.1), with 84.8% surviving at the end of follow-up (a total of 12 deaths; Figure 2). Survival did not differ by malignancy status (p = 0.624). Patients with echocardiographic signs of pulmonary hypertension (a tricuspid regurgitant jet velocity > 2.7 m/s) had lower survival (68.4% vs. 90.9%; p = 0.012; Figure 3). No survival difference was found regarding the use of methotrexate, mycophenolate mofetil, cyclosporine, rituximab, or cyclophosphamide. Intravenous methylprednisolone pulse therapy was associated with worse survival (75.8% vs. 90.9%; p = 0.02), likely reflecting more severe disease. A trend toward lower survival was seen in those with a > 10% decline in FVC (p = 0.06). 

## DISCUSSION

The present retrospective study of IIM-ILD patients followed at a referral center in Brazil found that females in the 50- to 59-year age bracket predominated, their diagnosis being delayed; dyspnea was the most common symptom-improving with treatment in most cases-together with extrapulmonary features such as muscle weakness and arthralgia; despite immunosuppression, 25.3% of the patients in the present study had a decline in FVC, whereas others remained stable or improved; echocardiographic signs of pulmonary hypertension were associated with worse prognosis; and methylprednisolone pulse therapy was associated with reduced survival, likely reflecting more severe disease. 

IIM has a global incidence of 5-10 per 100,000 adults and a prevalence of 14-17 per 100,000 adults.^7^ Polymyositis and dermatomyositis show a bimodal incidence peak: in childhood (at the age of 7 years, approximately) and adulthood (in the 30- to 50-year age bracket).^8^ IIM-ILD shows a clear female predominance, with 70-80% of cases occurring in women.^9^ In our study, 75.9% of the patients were women, with a mean age at diagnosis of 45.2 years, reflecting the typical profile of middle-aged female predominance reported in the literature.^10^ This pattern supports a role for hormonal, genetic, and immunological factors in female predisposition to IIM-ILD.^11^

Arthritis, mechanic’s hands and positivity for anti-ARS antibody (anti-Jo-1) have been identified as risk factors for a higher incidence of ILD.^3^ ^,^ ^12^ Our results highlight a high frequency of positivity for anti-Jo-1, which is a classic marker of ASyS and which is strongly associated with ILD. In our cohort, anti-Jo-1 was the most common of all anti-ARS antibodies, being found in 42 of 76 patients (55.3%), followed by anti-PL-12, in 7 of 77 (9.1%), and anti-PL-7, in 3 of 77 (3.9%). This finding is consistent with the literature, which shows that anti-Jo-1 is the most common autoantibody in this setting, followed by other, less common anti-ARS antibodies such as anti-PL-7 and anti-PL-12.^4^ ^,^ ^13^ These autoantibodies influence lung phenotype, disease severity, and treatment response, underscoring the role of serology in prognosis and risk stratification.^4^ ^,^ ^6^

The negative impact of ILD on survival is well documented in the literature, and the progression of ILD is represented functionally by a decrease in lung capacity.^14^ ^-^ ^16^ ILD is the main pulmonary manifestation of IIM/ASyS, contributing to morbidity and mortality, and may precede myositis in up to 20% of cases; early detection and treatment can be critical factors in patient prognosis.^17^ In our study, patients had a mean 17-month delay to diagnosis, potentially impacting ILD progression because of delayed treatment, and approximately 25% of patients had evidence of functional decline, with a decrease of > 10% in FVC. 

Initial IIM symptoms reflect the systemic inflammatory nature of IIM.^8^ Clinical severity closely correlates with autoantibody profile, aiding in guiding IIM/ASyS management.^18^ Non-muscular features such as fever, weight loss, skin involvement, gastrointestinal symptoms, ILD, arthritis, and Raynaud’s phenomenon are more common in patients who are positive for anti-ARS antibodies,^3^ and dyspnea, cough, hypoxemia, and reduced exercise capacity suggest pulmonary involvement.^19^ ^-^ ^21^ Dyspnea (in 94.9%), cough (in 70.8%), arthralgia (in 82.2%), and Raynaud’s phenomenon (60.7%) were common symptoms among our patients. These findings reinforce that respiratory symptoms, particularly dyspnea and cough, should prompt evaluation for pulmonary involvement in patients with rheumatologic diseases. 

Our study demonstrated spirometric stability or improvement in most patients, being consistent with prior studies. Correia et al. reported FVC stability/improvement in 60% of patients.^22^ Conticini et al. reported FVC stability/improvement in 100% of patients on mycophenolate mofetil/rituximab.^23^ González-Pérez et al. found > 10% increase in FVC in 67% of patients, especially with early treatment (< 6 months).^24^ These findings support the potential for functional improvement in IIM-ILD with timely and appropriate therapy. 

Pulmonary hypertension was suspected in 23.8% of patients. Right ventricular dysfunction was seen in only 1 patient, who required high-flow oxygen because of advanced parenchymal disease. Pulmonary hypertension was uncommon, and given that the ability of transthoracic echocardiography to estimate systolic pulmonary artery pressure accurately is often compromised in patients with fibrotic lung disease,^25^ this number of suspects is probably inaccurate. Current evidence supports the concept that although pulmonary hypertension is rare in IIM, it is linked to a worse prognosis when present.^26^ In the French pulmonary hypertension registry (n = 5,223), only 34 had IIM, with only three cases of IIM-associated pulmonary arterial hypertension.^27^ Most of those patients had pulmonary hypertension caused by parenchymal lung disease or overlap autoimmune syndromes.^27^ The findings of the present study are consistent with current evidence showing that pulmonary hypertension appears to be an indicator of advanced disease and carries a poor prognosis. We found no cases of pulmonary arterial hypertension in the present study. 

Initial IIM treatment typically includes oral or intravenous corticosteroid pulse therapy, although strong evidence of benefit is lacking.^28^ In our cohort, 98.7% received treatment with corticosteroids. Treatment generally involves corticosteroids, with or without immunosuppressants.^29^ ^-^ ^31^ However, the evidence for immunosuppressant use in IIM is based mainly on small open-label prospective studies and retrospective observational studies, which focus primarily on muscle strength, skin lesions, and functionality scales. Although the evidence for treating ILD in IIM is limited, the available evidence suggests a benefit from combining immunosuppressive drugs with corticosteroids in patients with IIM-ILD.^32^ ^-^ ^37^ Azathioprine, methotrexate, and mycophenolate mofetil were commonly used immunosuppressants, with no differences in outcomes. Intravenous methylprednisolone pulse therapy was reserved for severe cases, likely explaining their poorer prognosis. 

Bronchiectasis, reticulation, and honeycombing on CT suggest fibrosis and may occur in some IIM/ASyS patients.^38^ ^,^ ^39^ Antifibrotic agents such as nintedanib and pirfenidone are aimed at reducing the progression of the fibrotic component of ILD.^40^ In our study, none of the patients received treatment with antifibrotic agents, given that this class of drugs is not available for use in the Brazilian public health care system. 

The present study has limitations that are inherent to its retrospective design and its single-center nature, which may limit the generalization of the findings. The exclusion of 53 patients because of missing data was a significant loss and may have affected the results of the study, as well as having an impact on the frequency of missing data in the patients analyzed. Our study recruited patients with a diagnosis of IIM/ASyS and pulmonary involvement confirmed by chest CT. Because an extended antisynthetase antibody panel was not performed in all patients, it is possible that we missed some positive autoantibodies in our population. In patients in whom there was a high level of suspicion, the extended panel included antibodies such as anti-PL-7 and anti-PL-12. The fact that we had a convenience sample and that anti-Jo-1 is the most easily measured of all IIM-related autoantibodies makes the frequency of its positivity highly subject to selection bias. Additionally, the lack of pulmonary biopsy in most cases precludes a precise histopathological characterization of ILD, although HRCT scans were evaluated by experienced radiologists with expertise in ILD and, in clinical practice, lung biopsy is rarely performed in the context of autoimmune ILD. Plethysmography and DL_CO_ measurements were rarely performed in our patients, although they could have led to a better understanding of the physiological data during the follow-up period. Methylprednisolone pulse therapy was given to patients with greater initial severity; this probably explains the worse outcomes observed in those patients and represents a confounding bias. In addition, this association most likely does not represent a causal effect. However, the inclusion of a well-characterized cohort with a long follow-up period and serial functional data adds robustness to the analyses performed in the present study and enhances the clinical relevance of our results. 

The data from the present study are consistent with the literature in terms of epidemiology, serological profile of autoantibodies, clinical manifestations, frequency of CT findings, and survival outcomes. The analysis of functional behavior showed stability of or increase in FVC in most of the study participants with the combined use of immunosuppressants and oral corticosteroids, with no evidence of superiority of any drug. Mortality was higher in patients who received methylprednisolone pulse therapy and in those who had signs of pulmonary hypertension. 
